# Two Independent Acquisitions of Multidrug Resistance Gene *lsaC* in *Streptococcus pneumoniae* Serotype 20 Multilocus Sequence Type 1257 

**DOI:** 10.3201/eid3111.251101

**Published:** 2025-11

**Authors:** Bernard Beall, Wuling Lin, Zhongya Li, Theresa Tran, Benjamin J. Metcalf, Bridget J. Anderson, Keipp H. Talbot, Lesley McGee, Sopio Chochua

**Affiliations:** ASRT, Inc., Smyrna, Georgia, USA (B. Beall, Z. Li); Centers for Disease Control and Prevention, Atlanta, Georgia, USA (W. Lin, T. Tran, B.J. Metcalf, L. McGee, S. Chochua); New York State Department of Health, Albany, New York, USA (B.J. Anderson); Vanderbilt University School of Medicine, Nashville, Tennessee, USA (K.H. Talbot)

**Keywords:** antimicrobial resistance, bacteria, *Streptococcus pneumoniae*, clindamycin resistance, pneumococcal lineage, antimicrobial drugs, antimicrobial resistance, United States

## Abstract

Among >25,000 invasive pneumococcal disease isolates recovered in US locations during 2015–early 2024 through population-based surveillance, we detected 17 case isolates carrying the *lsaC* gene, which has been shown to confer resistance to clindamycin in group B *Streptococcus*. Sixteen isolates carried the *mef*, *msrD*, *tetM*, and *lsaC* genes on a 29-kb mobile element acquired through an interspecies recombination event and were intermediately clindamycin resistant. One isolate acquired a 62-kb mobile element containing the *ermB*, *tetM*, and *lsaC* genes through a transposition event. All 17 cases were in adults, including 4 adults experiencing homelessness and 9 with substance abuse problems. All 17 *lsaC*-positive isolates shared a 5.2-kb *lsaC-*containing element precisely integrated within the conserved *ori*T site of their respective mobile element. Those 17 *lsaC*-positive strains were all serotype 20, multilocus sequence type 1257, and were recovered recently (2021–2024); isolates 1–16 represented emergent disease clusters in New York and Connecticut.

*Streptococcus pneumoniae* is a leading pathogen globally and is the most common cause of community-acquired pneumonia, bacterial meningitis, bacteremia, and otitis media. Macrolides are often used to treat pneumococcal respiratory infections, and their frequent use has led to increases of macrolide resistance and coselected lincosamide resistance in *S. pneumoniae* (*1*). Although β-lactam antibiotics are first-line treatments, established guidelines exist for using intravenous clindamycin to treat community-acquired bacterial pneumonia (CABP) (*2*).

The 2 major macrolide-resistance mechanisms in streptococci are 23S rRNA methylation by *erm* gene–encoded methylases (confers resistance to macrolides, lincosamides, and streptogramin B antibiotics) and removal of macrolides by the *mef/msrD*-encoded efflux pumps. In group B *Streptococcus* (GBS), 2 additional mechanisms conferring lincosamide resistance have been described: lincosamide modification by nucleotidyltransferases (*lnu* genes) (*3*,*4*), and the resistance to lincosamides, streptogramin A, and pleuromutilins conferred by the *lsaE*- or *lsaC*-encoded ATP-binding cassette (ABC) transporters (*5*,*6*). Streptogramin antibiotics have been used for skin infections caused by gram-positive species, and although pleuromutilins are primarily used for veterinary purposes (*7*), use of the pleuromutilin lefamulin has been approved by the US Food and Drug Administration for systemic treatment of CABP in adults (*8*). In a recent study (*9*), 211 strains of *S. pneumoniae* tested, including 105 that were penicillin resistant, were all lefamulin susceptible according to Clinical and Laboratory Standards Institute criteria (*10*), with MICs <0.25 µg/mL. In this study, we describe acquisitions of a conserved *lsaC*-containing element in pneumococci that appear to have been introduced through 2 independent mechanisms of horizontal transfer into the same serotype 20, multilocus sequence type (ST) 1257 (serotype 20/ST1257) strain lineage.

## Materials and Methods

### Active Bacterial Core Surveillance Data

We defined invasive pneumococcal disease (IPD) cases through isolating *S. pneumoniae* from a normally sterile site in an Active Bacterial Core surveillance (ABCs) (*11*) area resident. The surveillance sites included entire US states or selected counties in 10 states, representing ≈30–35 million persons during 2015–2024 (*11*). We reviewed case medical charts to obtain demographic information. We defined adults experiencing homelessness as adults documented as homeless or residing in a shelter, mission, medical respite, or church community center at the time of positive culture. This activity was reviewed by a Centers for Disease Control and Prevention (CDC) review board, was deemed not to be human participants research, and was conducted consistent with applicable federal law and CDC policy. ABCs sites obtained ethics approval from their state health department and academic partner institutional review boards as necessary.

### ABCs Isolate Characterization

Isolates were assigned serotypes, multilocus STs, and resistance features employing short-read whole-genome sequencing as previously described within the CDC Pneumonia and Streptococcus Laboratory Branch (National Center for Immunization and Respiratory Diseases, Division of Bacterial Diseases) (*12*,*13*). We deposited genome sequences in the National Center for Biotechnology Information Sequence Read Archive (BioProject no. PRJNA284954). We used broth microdilution testing in the Pneumonia and Streptococcus Laboratory Branch as previously described (*14*) to verify resistance phenotypes predicted for clindamycin, erythromycin, tetracycline, and tiamulin. Tiamulin has the same mechanism of action as lefamulin, and the 2 antimicrobial drugs share closely similar MICs for pneumococci and for bacterial in vitro transcription-translation (*15*). Pneumococcal susceptibility to lefamulin corresponds to a MIC of <0.5 µg/mL according to Clinical and Laboratory Standards Institute guidelines (*10*).

### Long-Read Sequencing

Long-read sequencing was conducted by the CDC Biotechnology Core Facility Branch (Office of Laboratory Systems and Response, Division of Core Laboratory Services and Response) to provide a single contig genome resource of a representative *lsaC-*positive isolate and to map the *lsaC* element; the PacBio Microbial Multiplexing procedure (Pacific Biosciences, https://www.pacb.com) was implemented. Genomic DNA extracted for short-read sequencing was also used to generate a representative single contig genome (isolate identifier 20234456). We generated libraries using the SMRTbell Express Template Prep Kit 3.0 and size-selected using a 0.45x Ampure bead cleanup (Pacific Biosciences) to remove small DNA (<5 kb). We sequenced final libraries for 30 hours after 30 minutes preextension times on the Sequel II using Sequel II Binding Kit 3.2 (Pacific Biosciences). We used PacBio HiFi reads in Flye v2.9 (*16*,*17*) to conduct de novo assembly for the genome size of 2.13 Mb and evaluated the assembled circularized contig by using BLAST + 2.9.0 (*18*). A coverage of 367 was obtained for the strain 20234456 genome, which we deposited into GenBank (accession no. CP178339).

### Genomic Analyses

We used Prokka version 1.14.5 (*19*) to obtain annotated open reading frames and then used EasyFig version 2.2.3 (*20*) to align sequences and generate figures. We generated core-genomic maximum parsimony trees and pairwise single-nucleotide polymorphism (SNP) matrices from short-read bacterial genome sequences using kSNP3.0 with a kmer size of 19 (*21*). We used resultant core.tre files generated from kSNP3.0 to generate core-genomic pairwise SNP comparisons and phylogenetic diagrams using MEGA7 (*22*). We used the Proksee website (*23*) to generate a circular map of a representative *lsaC*-positive isolate and to predict mobile element genes using the mobileOG-db database (*24*). We used progressive Mauve (*25*) to generate aligned core genomes of representative serotype 20/ST1257 isolates and subjected core genome alignments to Gubbins (*26*) to identify recombinant regions.

## Results

### Serotype 20/ST1257 Invasive Pneumococcal Disease Isolate Phylogeny

 Genomic sequence–based ABCs strain surveillance carried out during 2015–2023 (3 serotype 20/ST1257 isolates [1 *lsaC*-positive] were also included from 2024) included >25,000 genomes from invasive pneumococcal disease (IPD) case isolates recovered during this period. Of those, 17 IPD isolates recovered during 2020–early 2024 tested positive for the *lsaC* determinant (Table). All 17 *lsaC*-positive isolates were serotype 20/ST1257, were recovered from adults 34–84 years of age, and were among 367 serotype 20/ST1257 IPD isolates recovered during 2015–early 2024.

**Table:** Selected antibiotic phenotype testing for serotype 20 ST1257 *lsaC*-positive and control isolates in study of 2 independent acquisitions of multidrug resistance gene *lsaC* in serotype 20 ST 1257 *Streptococcus pneumoniae* isolates, United States*

Isolate no.†	Laboratory ID no.	Resistance genotype	MIC, µg/mL (resistance)‡					State
			ERY	CLI	LEF§	TIA¶	TET	
C	ATCC49619	Negative	<0.12 (S)	<0.12 (S)	<0.25 (S)	<0.5	<0.25 (S)	NA
C	20195631	Negative	<0.06 (S)	0.12 (S)	ND	ND	<0.25 (S)	NA
C	20236374	*ermB*	4 (R)	>2	<0.25 (S)	1	<0.25 (S)	NA
17	20204687	*ermB*, *lsaC*, *tetM*	4 (R)	>2	<0.25 (S)	1	>8 (R)	TN
1–3	20228033, 20238465, 20246104	*lsaC*, *mef/msrD*, *tetM*	2–8 (R)	0.5 (I)	0.5 (S)	2	>8 (R)	NY
4	20242307	*lsaC*, *mef/msrD*, *tetM*	2–8 (R)	0.5 (I)	0.5 (S)	1	>8 (R)	CT
5–11	20223565, 20232253, 20232771, 20235035, 20235950, 20241474, 20243458	*lsaC*, *mef/msrD*, *tetM*	2–8 (R)	0.5 (I)	<0.25 (S)	2	>8 (R)	NY (5), CT (2)
12–15	20212635	*lsaC*, *mef/msrD*, *tetM*	2–8 (R)	0.5 (I)	<0.25 (S)	1	>8 (R)	CT (2), NY (2)
	20234456							
	20237578							
	20244259							
16	20232812	*lsaC*, *mef/msrD*, *tetM*	2–8 (R)	0.5 (I)	<0.25 (S)	<0.5	>8 (R)	NY

*C, negative control; CLI, clindamycin; CLSI, Clinical Laboratory Standards Institute; CT, Connecticut; ERY, erythromycin; I, intermediately resistant; ID, identification; LEF, lefamulin; NA, not applicable; ND, not done; R, resistant; S, susceptible; ST, sequence type; TET, tetracycline; TIA, tiamulin. †Isolates 1–17 correlate to isolates described in text. All isolates listed are from Active Bacterial Core surveillance and are serotype 20/ST1257, except for ATCC49619. ‡Susceptible, intermediately resistant, or resistant as described in CLSI (*10*). §LEF susceptibility is defined by CLSI as <0.5 µg/ml (*10*). Resistance values are currently undefined. ¶TIA susceptibility breakpoints have not been defined.

**Figure 1:**
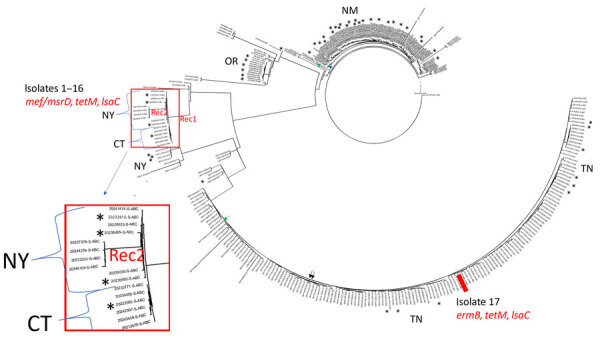
Core-genomic phylogeny of serotype 20/ST1257 invasive pneumococcal disease isolates recovered during 2015–2024 in study of 2 independent acquisitions of multidrug resistance gene *lsaC* in serotype 20/ST1257 *Streptococcus pneumoniae*, United States. Of 367 serotype 20/ST1257 invasive pneumococcal disease isolates recovered during 2015–2024, we included 358 with <200 short-read genome contigs in the phylogram. Only 3 year 2024 isolates, including 1 of the depicted *lsaC*-positive isolates 1–16, were included. There were 6,727 variable positions. Isolates 1–16 are indicated on a single branch with the red box divided into 2 subclusters specific to New York (10 isolates) and Connecticut (6 isolates) Active Bacterial Core surveillance sites. The 10-isolate New York subcluster has an inner subbranch of 6 isolates and outer subbranch of 4 isolates. The main branch containing isolates 1–16 and the outward New York subbranch are labeled with Rec, indicative of the disproportionate contribution of recombination to the long branch lengths. Asterisks indicate 50 isolates from adults experiencing homelessness. The remaining isolates were susceptible to antibiotics, except for 2 *mef/msrD*-positive isolates (green arrows), 2 *tetM*-positive isolates (black arrows), and 1 *ermB*-positive isolate (blue arrow). Isolate 17 (positive for *ermB*, *tetM*, *lsaC*) is indicated with a solid red rectangle. ST, sequence type.

A single *lsaC*-positive isolate (isolate 17) recovered in Tennessee was *ermB*+/*tetM+* and was phylogenetically distinct from *lsaC*-positive isolates 1–16 recovered in New York and Connecticut that were positive for the *mef*/*msrD* macrolide efflux genes and *tetM* (Figure 1). The phylogenetic tree revealed 2 separate geographic clusters corresponding to New York and Connecticut. Twelve genomes (representing all 6 Connecticut isolates and 6 New York isolates) revealed a range of 2–20 pairwise SNP differences; the average was 11 SNPs. The genomes from 4 New York isolates recovered during 2022–2024 formed a separate subcluster (Figure 1); the pairwise SNP difference range was 5–14 SNPs (average of 9.5 SNPs).

### Resistance Phenotypes

Isolate 17 was fully resistant to clindamycin (MIC >2 µg/mL) and erythromycin (MIC 4 µg/mL). Isolates 1–16 shared the clindamycin MIC of 0.5 µg/mL and erythromycin MICs of 2–8 µg/mL, compared with MICs of <0.12 µg/ml for clindamycin and erythromycin in control serotype 20/ST1257 isolate 20195631 and strain ATCC49619 (Table).

All 17 *lsaC*-positive isolates tested as susceptible to lefamulin (MIC range of <0.25–0.5 µg/mL). However, isolates 1–3 revealed MICs to both pleuromutilins that were above control values, and isolates 4–11 revealed MICs to 1 pleuromutilin that were above control values. Although inconclusive, those data are consistent with the *lsaC* gene conferring reduced susceptibility to pleuromutilins compared with *lsaC-*negative strains, especially when considering that the *lsaC* determinant in isolates 1–16 appears to encode an active clindamycin efflux component conferring intermediate clindamycin resistance. Besides isolates 1–17, 5 serotype 20/ST1257 isolates carried resistance determinants, consisting of a single *ermB*-positive isolate, 2 *mef/msrD*-positive isolates, and 2 *tetM*-positive isolates, none of which were phylogenetically related to isolates 1–16 or isolate 17 (Figure 1).

### Mobile Element Carrying Isolate 17 *lsaC* Gene

**Figure 2:**
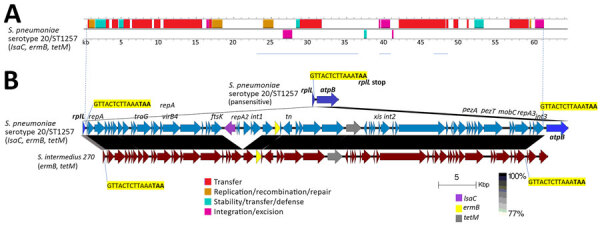
Complex mobile element within isolate 17 (positive for *ermB*, *tetM*, and *lsaC*) in study of 2 independent acquisitions of multidrug resistance gene *lsaC* in serotype 20/ST1257 *Streptococcus pneumoniae*, United States. A) Predicted mobile element genes and functions. B) The insertion site is depicted between *rplL* and *atpB* in pansusceptible genome serotype 20/ST1257 with *rplL* 3′ 15 bp target site highlighted in yellow. The alignment between corresponding genomic regions of isolate 17 and *S. intermedius* strain 270 is shown with 15 bp repeat of *rpllL* 3′ end indicated upstream of genomic *atpB* gene in isolate 16 and upstream of unidentified open frames in *S. intermedius* 270. Although the *S. intermedius rplL* gene had an identical 15-bp terminus, it only shares 90% sequence identity with the pneumococcal *rplL* gene. The original EasyFig (*20*) output was modified to depict the interspecies homology between the 2 *rplL* homologs. Homology legend indicates range of 77%–100% sequence identity, depicting ≈90% sequence identity between the 2 different *rplL* alleles and >96% sequence identity between the 2 insertion elements. ST, sequence type.

Examination of the region carrying the *lsaC* gene in isolate 17 revealed a complex 61-kb mobile element of the broad Tn916/Tn1545 conjugative transposon family (*27*) inserted within the 3′ end of the ribosomal protein gene *rplL* (corresponding to base 1,215,206 of the Tigr4 reference genome [GenBank accession no. CP000410]). That insertion revealed evidence of a transposition event shown by the tandem repeat of the 3′ 5 codons of the *rplL* gene at the distal end of the 61-kb element (Figure 2). That element shared its highest similarity to an element from *S*. *intermedius* (GenBank accession no. CP003858) with >98% sequence identity over the overlap (Figure 2). The *S. intermedius* insertion was also within the corresponding site of its *rplL* gene that shared 90.8% sequence identity with the isolate 17 *rplL* gene.

### Mitis Group Streptococcal Source of Mobile Element Carried by Isolates 1–16

**Figure 3:**
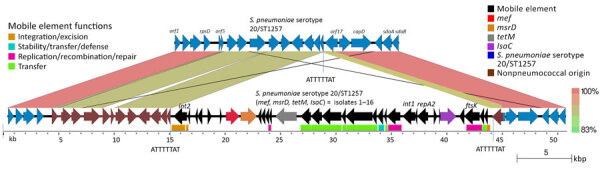
Introduction of isolate 1–16 mobile element into *Streptococcus pneumoniae* serotype 20/ST1257 by a double crossover homologous transformation event in study of independent acquisitions of multidrug resistance gene *lsaC* in serotype 20/ST1257 *S. pneumoniae* isolates, United States. Crossover 1 occurred within *orf5* and crossover 2 occurred near the 5′ end of *orf17.* Apparent nonpneumococcal homolog genes in isolates 1–16 (serotype 20/ST1257 [*mef*, *msrD*, *tetM*, *lsaC*]) flanking the mobile element (*orf* *5–16*) are indicated upstream in brown, as is most of *orf17* immediately downstream of the insertion element. These genes share <90% sequence identity with the parental strain lineage serotype 20/ST1257. The section corresponding to *orf17* has been modified from the original EasyFig (*20*) depiction to reflect more accurately the decreased homology between the 2 *orf17* alleles and the actual breakpoint in homology. The repeated 8-bp target site for the ancestral transposition event is indicated. Predicted mobile element genes and functions are indicated in the below grid corresponding to 15–44.2 kb of the entire isolate 1–16 region. *orf5* is indicated by 1 open reading frame in isolates 1–16 but is indicated by 2 short homologous reading frames in the parental strain (top). ST, sequence type.

**Figure 4:**
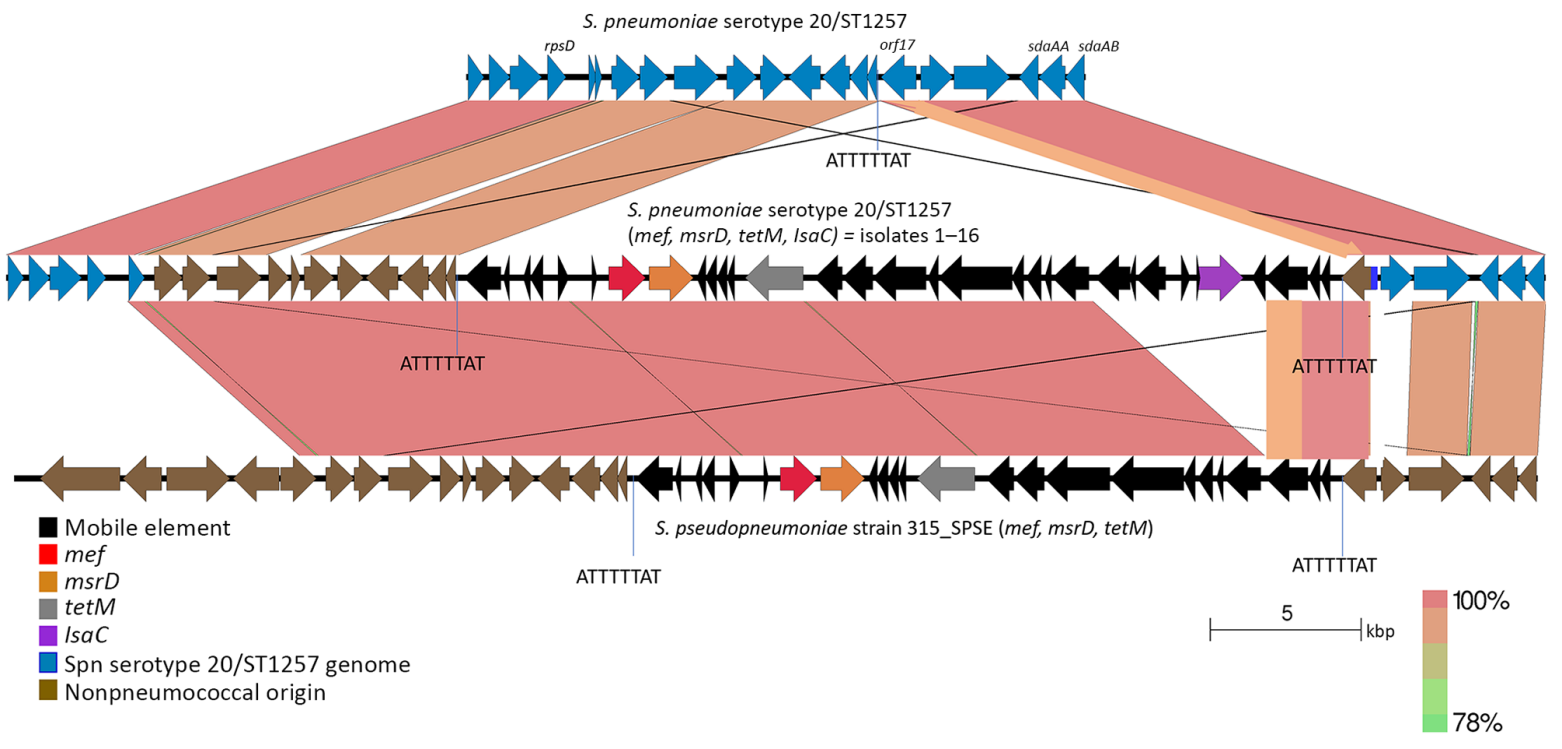
Near sequence identity shared between pneumococcal isolates 1–16 and *Streptococcus pseudopneumoniae* strain 315_SPE in mobile element insertion region in study of 2 independent acquisitions of multidrug resistance gene *lsaC* in serotype 20/ST1257 *S. pneumoniae*, United States. The near-identical region includes much of the mobile element itself, and flanking genes that diverge from pneumococcal parental recipient strain (top). The EasyFig (*20*) output homology was modified to reflect boundaries between marked homology differences between the 2 pneumococcal strains (focused upon *orf17* only) and between the middle pneumococcal strain and the below *S. pseudopneumoniae* strain (encompassing the last 3 *orf*s of the mobile elements and most of *orf17*). ST, sequence type.

**Figure 5:**
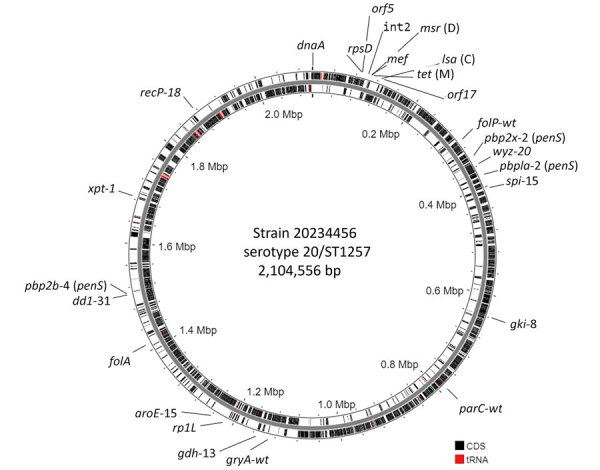
Single contig genome of isolate 20234456 (*msrD*-positive, *mef*-positive, *tetM*-positive, *lsaC*-positive) representative of isolates 1–16 in study of 2 independent acquisitions of multidrug resistance gene *lsaC* in serotype 20/ST1257 *Streptococcus pneumoniae*, United States. Position 1 starts at the *dnaA* structural gene. CDSs and tRNA genes are indicated on the forward strand (outer band) and reverse strand (inner band). The genes approximating the location of the interspecies double-crossover event introducing the resistance element lie between the *rpsD* ribosomal protein gene and the putative transport gene *orf17*. The ribosomal protein gene *rplL* is also depicted near the 1.2-Mbp marker to show the approximate genomic location of the accessory element carrying the *ermB*, *tetM*, and *lsaC* determinants in isolate 17. Other determinants shown reflect potential genomic resistance determinants (all indicative of susceptibility in this genome according to bioinformatic pipeline output), including *folP*/*folA* (fluoroquinolones); *parC*/*gyrA* (fluoroquinolones); and *pbp2x/pbp1a/pbp2b* (β-lactam antibiotics). In addition, the multilocus sequence typing markers *spi-15*, *gki-8*, *aroE-15*, *ddl-31*, *xpt-31*, and *recp-18* define ST1257. CDS, coding sequences; ST, sequence type.

**Figure 6:**
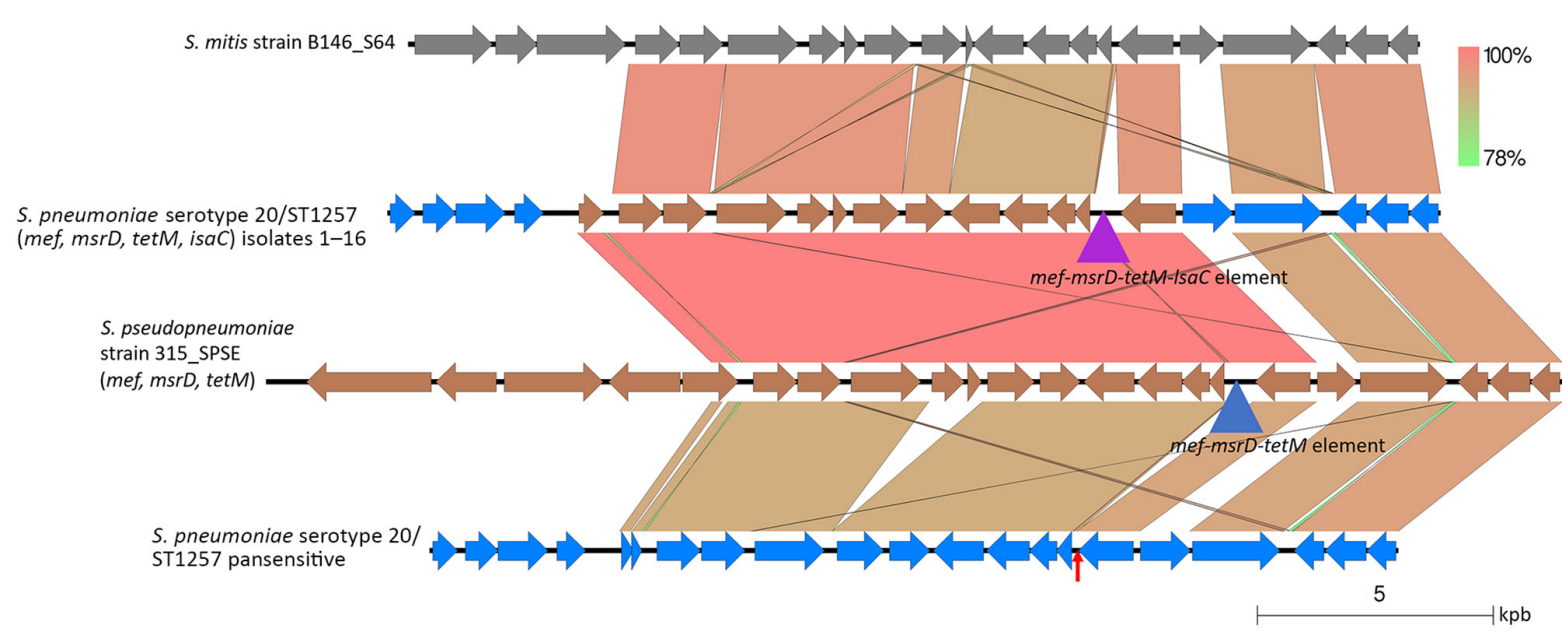
Alignments between *Streptococcus mitis* strain, pneumococcal isolates 1–16, *S. pseudopneumoniae* strain 315_SPSE, and pansusceptible serotype 20/ST1257 pneumococcal strain (bottom) in study of 2 independent acquisitions of multidrug resistance gene *lsaC* in serotype 20/ST1257 *S. pneumoniae*, United States. This alignment depicts representative homology between certain streptococcal strains recognized as *S. mitis* and the genome flanking the resistance element in strains 1–16. Also shown is the near identity to this region shown by *S. pseudopneumoniae* strain 315_SPSE. The mobile elements (triangles) in isolates 1–16 and *S. pseudopneumoniae* 315_SPSE correspond to the mobile elements depicted in Figure 4. The apparent nonpneumococcal genes (brown) flank the mobile element introduced by recombination into isolates 1–16. Red arrows indicates the genomic region in the serotype 20/ST1257 corresponding to the mobile element insertion site. ST, sequence type.

**Figure 7:**
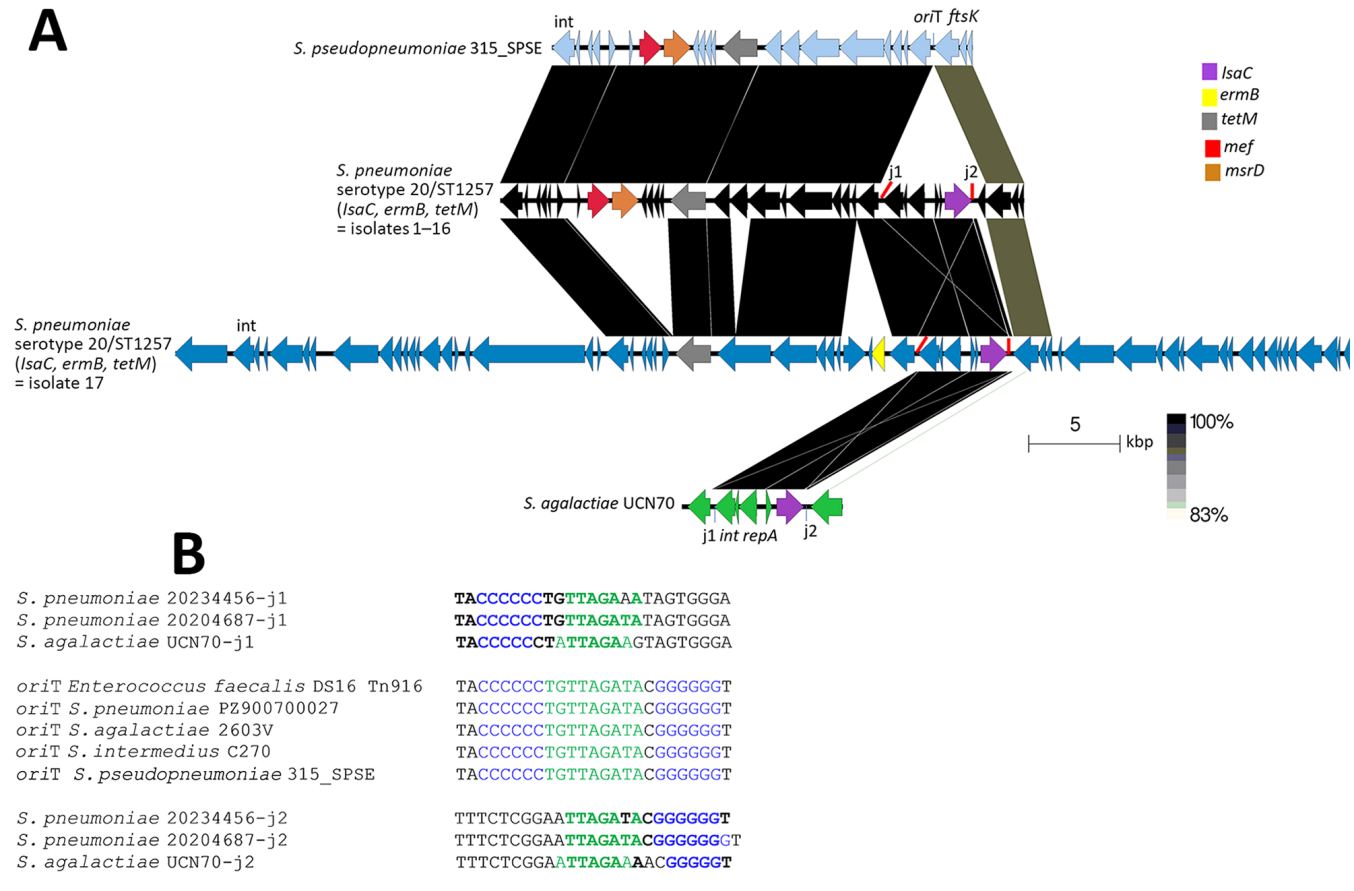
Related regions shared between the 2 pneumococcal *lsaC* elements from this study, the mobile element found in *S. pseudopneumoniae* 315_SP_SPSE, and the partial mobile element from GBS strain UCN70 in study of 2 independent acquisitions of multidrug resistance gene *lsaC* in serotype 20/ST1257 *S. pneumoniae*, United States. A) The *lsaC* element target site (*ori*T) is indicated in *S. pseudopneumoniae* 315_SPSE, with partially homologous junctions (j1 and j2) of the *lsaC* elements in the 3 strains (isolate 1–16, isolate 17, and *S. agalactiae* UCN70). B) The identical *ori*T sequence shared between related mobile elements lacking *lsaC* from 5 different species is shown. *lsaC* element junction j1 shares homology with the 5' *ori*T sequence and junction j2 shares homology with the 3′ *ori*T sequence. Purple indicates the shared C and G repeats; green indicates direct repeats between each of the 3 strains shared between their specific j1 and j2 sequences and homologous central *ori*T sequence. Bold indicates J1 and j2 bases shared with the *ori*T sequence. ST, sequence type.

Isolates 1–16 carried an identical 41-kb region that consisted of a 29-kb mobile element flanked by ≈12 kb of upstream and 1 kb of downstream divergent chromosomal sequence that shared 88% sequence identity with the corresponding region of serotype 20/ST1257 pansusceptible isolates (Figure 3). This flanking region and the predicted mobile element shared >99% sequence identity to its closest match in GenBank, *S*. *pseudopneumoniae* strain 315_SPSE (accession no. NZ_JVLX00000000) (Figure 4). The upstream crossover in this presumed homologous recombination event occurred just downstream of ribosomal protein gene *rpsD* (position 89,092 within *lsaC*-positive serotype 20/ST1257 strain 20234456) (Figure 5); the downstream crossover point corresponded to base 130,195 within putative transport protein gene *orf17*. Those positions corresponded to base 86,400 (upstream crossover) and 97,643 (downstream crossover) of the Tigr4 reference genome. *S. pseudopneumoniae* strain 315_SPSE was an outlier, in that the next 10 closest matches to the 12-kb flanking sequences, although representative of *S. pseudopneumoniae* and *S*. *mitis* strains, shared only ≈95% sequence identity to the 12-kb flanking sequence (Figure 6). The predicted 29-kb mobile element from isolates 1–16 shared high similarity in its overlap with the mobile element in isolate 16 except for presence of the 5.3 kb *mef/msrD* region and lack of the 2 kb *ermB* region (Figure 7, panel A).

### 5255-bp Integrative *lsaC* Element Shared by *lsaC*-Carrying Isolates

All 17 *lsaC*-positive isolates carried the complete 1,479-bp *lsaC* gene as part of a 5,255-bp integrative element also conserved with a counterpart locus in the GBS strain UCN70 (*5*) and predicted to carry genes encoding a site-specific integrase and a replication initiation protein (Figure 7, panel A). The 5,255-bp isolate 1–16 *lsaC* element differed by 5 bp from isolate 17 and 10 bp from GBS UCN70. The isolate 1–16 *lsaC* allele differed from the isolate 17 *lsaC* allele by 2 missense substitutions, and those 2 alleles differed from GBS UCN70 *lsaC* allele by 1–2 missense substitutions. As described for related *lsaC* elements (*6*), each 5,255-bp element contained flanking homologous junctions (j1 and j2 in Figure 7, panel B) that shared repeated regions after a run of 6 Cs (j1) or preceding a run of 5–7 Gs (j2). Junctions j1 and j2 each shared overlapping sequence nearly identical to an *ori*T sequence motif shared by various related conjugative transposons, indicative of specific integration of the 3 different 5,255-bp *lsaC* elements within transposons harboring an identical *ori*T (Figure 7, panel B).

### Features of Serotype 20/ST1257 Cases

Only 9 of the 367 serotype 20/ST1257 case-patients were <18 years of age; only 1 patient was <5 years of age. Of the 357 case-patients with known residence status, 50 (14.0%) were adults experiencing homelessness, including 4 (23.5%) of the case-patients whose cases corresponded to 4 of the isolates 1–17. This finding is consistent with data from ABCs in 2018 and 2019 (*28*,*29*), in which serotype 20 made up the third highest proportion of cases among adults experiencing homelessness, surpassed only by serotypes 4 and 12F. Of serotype 20 cases, 14.9% were from adults experiencing homelessness in 2018 and 12.2% were from adults experiencing homelessness in 2019. Typical of serotypes more common among adults experiencing homelessness (*29*), 9 of the 17 serotype 20 *lsaC*-positive isolates were recovered from adults with substance use problems involving cocaine or methamphetamines (6 persons, 1 of which was an adult experiencing homelessness) or alcohol (2 persons).

### Contribution of Interspecies and Intraspecies Recombination to Apparent Phylogenic Distance of Isolates 1–16 from Closest Pansensitive Ancestor

To assess temporal distances between *lsaC*-positive genetic progeny and their closest ancestors, we generated a core-genome phylogeny of serotype 20/ST1257 IPD isolates (Figure 1). Isolate 17 differed by only 12 SNPs from the closest pansensitive strains (Figure 1). That finding is consistent with a recent transposition event in which the mobile element was precisely inserted within the genomic *rplL* gene (Figure 2). In contrast, the inner subbranch separating isolates 1–16 from its predicted most common ancestor showed a distance of ≈130 SNPs. The distance between closest pansensitive isolates (2 isolates recovered in Connecticut during 2015–2018) and the subcluster of 6 New York isolates within isolates 1–16 was 140–180 SNPs. Through recombination analysis of aligned genomes (*26*), all or most of this pairwise distance (152 SNPs) was introduced into the depicted phylogeny (Rec1 in Figure 1) by the single interspecies recombination event that introduced the resistance element along with ≈13 kb of nonpneumococcal flanking core-genomic sequence (Figures 3–5).

The 10 New York isolates (all recovered during 2022–2024) were additionally subdivided into 2 subclusters by 5 recombinant fragments uniformly present in the distal 4 isolates on the tree (data not shown). The most distal subcluster (consisting of isolates recovered during 2022–2024) displayed considerable distance (104–117 SNPs) from the other 6 New York isolates (recovered during 2022–2023). The 5 recombinant regions within the 4 distal subcluster isolates ranged from 750–9,020 bp in length and accounted for all or most of this entire SNP distance between the 2 subgroups (Rec2 in Figure 1); that finding was indicative of a close temporal distance between those 4 isolates and the 6 ancestral New York isolates.

## Discussion

*lsaC* determinants have been described from GBS (*5*,*6*) and very recently have been reported from animal strains of *S. equisimilis* (*30*). In this study, we identified 2 independent introductions of *lsaC* into pneumococci where the determinant is present on 2 distinct mobile elements, 1 cocarrying *ermB* and *tetM*, and 1 cocarrying *mef*, *msrD*, and *tetM*. Within both mobile elements, the *lsaC* component element targeted *ori*T through a conserved mechanism first described in GBS (*6*). Within isolate 17, the large mobile element carrying *ermB* and *tetM* was likely to have been first introduced into the genome through transposition targeting the *rplL* 3′ 15-bp target, followed by *ori*T-targeted insertion of *lsaC*. Disruption of the *ori*T sequence would presumably prevent further high-frequency transposition of the composite transposon containing *ermB*, *tetM*, and *lsaC*. Because the mobile element observed within isolates 1–16 was introduced through interspecies homologous transformation (*31*) and not by conjugative transposition requiring an intact *ori*T, predicting whether the *lsaC* element was cocarried with other resistance genes or introduced subsequently is not possible. These 2 *lsaC* elements did not recently originate from the same donor bacterial strain; they differed by 5 SNPs, including 2 missense substitutions within the *lsaC* gene. The apparent *ori*T-targeting mechanism of *lsaC* horizontal transfer has the potential to include numerous bacterial species because of the ubiquity of conjugative elements sharing the *ori*T target for *lsaC* element insertion and the presence of transposition genes (site specific integrase/replication initiation) genes within the *lsaC* element itself.

Serotype 20 is one of the few serotypes that have an extremely high propensity for generating genomic clusters among IPD isolates recovered from adults, where a so-called clustering isolate differs from >1 isolates by <10 SNPs (*28*,*29*,*32*,*33*). The 23-valent pneumococcal polysaccharide vaccine introduced in 1983 contains a serotype 20 component; however, the immunity conferred by this vaccine decreases within a few years (*34*). Despite the introduction of highly effective pneumococcal protein-polysaccharide conjugate vaccines, starting with the 7-valent vaccine (PCV7) in 2000, serotype 20 was not included within another licensed vaccine until the licensure of PCV21 in 2024 for adults 19–64 years of age with risk conditions for IPD and for those >65 years (*35*). The serotypes that have the highest proportions of clustering IPD isolates, such as 4, 12F, and 20, have the greatest propensity to cause IPD among adults experiencing homelessness and are rarely found in carriage (*35*–*39*). Those serotypes have been characterized as short carriage–duration serotypes (*40*) and rapidly spread between adults, causing IPD in vulnerable hosts shortly after acquisition in carriage (4*0*,*41*). Serotypes such as 4, 12F, and 20 differ from serotypes often found in pediatric carriage, such as 35B and 19A (*35*,*36*) that are associated with relatively low frequencies of genomic clustering (*29*). The prevalence in IPD of such long duration–carriage serotypes as 35B and 19A appear directly related to their higher detection incidence in pediatric carriage (*35*–*37*,*39*); that observation is supported by the well-characterized herd effect of adult IPD prevention affected by high coverage of conjugate vaccines among the pediatric population (*41*,*42*).

Serotype 4, which has the highest propensity for both genomic clusters and causing IPD among adults experiencing homelessness, was recently found to acquire reduced susceptibility to vancomycin through the acquisition of a *vanG-*containing mobile element (*14*). The acquisitions of *vanG* and *lsaC* in pneumococci had not been reported before those very recent observations of their presence in the short carriage–duration serotypes. Another recent and unusual finding within those high-clustering serotypes was the generation of a serotype 12F to serotype 4 serotype-switch variant IPD cluster causing multiple IPD cases among adults experiencing homelessness (*32*). Recent increases (*45*) in the number of persons lacking permanent shelter (record numbers of 653,104 persons in 2023 and 771,480 persons in 2024) are likely to directly correlate to concurrent increases of IPD caused by high-clustering serotypes among adults experiencing disadvantages (*28*,*29*,*32*,*33*,*45*,*46*). Those increases might have contributed to unusual interspecies and intraspecies horizontal genetic transfer events involving those serotypes.

The acquisition of *lsaC* in isolate 17 is of potential medical significance, even though the strain is already fully clindamycin resistant through the presence of *ermB* on the same mobile element. Even though streptogramin A and pleuromutilin antibiotics are currently used primarily for veterinary purposes (*7*), the pleuromutilin lefamulin was recently approved for systemic use in cases of CABP in adults (*8*). Although all 17 isolates in this study were susceptible to lefamulin, 4 tested at the breakpoint of susceptibility, and 10 revealed MICs that were above control values for the closely related pleuromutilin tiamulin. In view of the cross-resistance to clindamycin and pleuromutilins observed in GBS, it is reasonable to assume *lsaC-*conferred intermediate clindamycin-resistance and to suspect reduced pleuromutilin susceptibility in isolates 1–16. In isolates 1–16, the presence of *lsaC* would prevent the use of clindamycin for certain situations, such as strain penicillin resistance or patient allergy in CABP cases. The physical linkage of *lsaC* with other resistance genes also provides the obvious potential for coselection of the unrelated resistance determinants in bacterial strains.

Of note, isolates 1–16 appear to have recently emerged and are actively expanding within 2 different ABCs sites; the most recent isolate appeared within the small proportion of 2024 ABCs strain surveillance completed at that time. The apparent temporal distance shown between the isolate 1–16 lineage and pansusceptible serotype 20/ST1257 isolate is mainly artifactual because of SNPs introduced through recombination at the *lsaC* insertion region of the genome. This *lsaC*-positive cluster has continued to recently diversify; a 4-isolate subcluster revealed multifragment recombination, which appears to commonly occur in pneumococci from a single exposure to donor DNA (*32*,*48*).

The recent acquisitions of *lsaC* and *vanG* within invasive pneumococci highlight the granular nature of whole-genome sequence–based disease strain surveillance for detecting rare resistance gene acquisition events and for monitoring subsequent spread of such novel strains. With continued narrowing of available antibiotic choices for treating bacterial infections, maintaining this vigilance through long-term surveillance of invasive strains is key.
